# Decompressive hemicraniectomy versus medical treatment of malignant middle cerebral artery infarction: a systematic review and meta-analysis

**DOI:** 10.1042/BSR20191448

**Published:** 2020-01-06

**Authors:** Heng Wei, Fu-Min Jia, Hong-Xiang Yin, Zhen-Li Guo

**Affiliations:** Department of Neurology, Hubei Provincial Hospital of Integrated Chinese and Western Medicine, Hubei University of Chinese Medicine, Wuhan 430000, China

**Keywords:** decompressive hemicraniectomy, malignant infarction of the middle cerebral artery, medical treatment, randomised controlled trials

## Abstract

**Objectives:** To estimate evidence for decompressive hemicraniectomy (DHC) versus medical treatment effects on survival rate and favorable functional recovery among patients of malignant middle cerebral artery infarction (MMCAI) in randomized controlled trials (RCTs).

**Design:** The present study is a systematic review and meta-analysis of RCTs.

**Setting:** The MEDLINE/PubMed, EMBASE, Springer, Cochrane Collaboration database, China National Knowledge Infrastructure (CNKI) database, and Wanfang database were comprehensively searched for RCTs regarding the effects of DHC versus medical treatment among patients of MMCAI in these English and Chinese electronic databases from inception to 1 June 2019. Two reviewers independently retrieved RCTs and extracted relevant information. The methodological quality of the included trials was estimated using the Cochrane risk of bias tool. Review Manager5.3.5 software was used for statistical analyses. The statistical power of meta-analysis was estimated by Power and Precision, version 4 software.

**Participants:** Nine RCTs with a total of 425 patients with MMCAI, containing 210 cases in the DHC group and 215 cases in the medical treatment group, met the inclusion criteria were included. Primary outcomes were measured by survival rate, defined as modified Rankin scale (mRS) score 0–5 and favorable functional recovery as mRS score 0–3. The follow-up time of all studies was at 6–12months.

**Results:** First, compared with the medical treatment group, DHC was associated with a statistically significant increase survival rate (RR: 1.96, 95%CI 1.61–2.38, *P* < 0.00001) and favorable functional recovery (RR: 1.62, 95%CI 1.11–2.37, *P* = 0.01). Second, subgroup analysis: (1) Compared with the medical treatment group among patients age ≤60 years, DHC was associated with a statistically significant increase survival rate (RR = 2.20, 95%CI 1.60–3.04, *P* < 0.00001); (2) Compared with the medical treatment group among patients of age >60 years, DHC was also associated with a statistically significant increase survival rate (RR: 1.93, 95%CI 1.45–2.59, *P* < 0.00001); (3) Compared with the medical treatment group, the time of DHC was preformed within 48 h from the onset of stroke that could statistically significant increase survival rate (RR: 2.16, 95%CI 1.69–2.75, *P* < 0.00001). Third, sensitivity analyses that measured the results were consistent, indicating that the results were stable. Fourth, the results of statistical power analysis were ≥80%. Finally, the funnel plot of the survival rate included nine RCTs showed no remarkable publication bias.

**Conclusions:** Our study results indicated that DHC could increase survival rate and favorable functional recovery among patients age ≤60 or >60 years. The optimal time for DHC might be no more than 48 h from the onset of symptoms. However, due to the limitations of this research, it is necessary to design high quality, large-scale RCTs to further evaluate these findings.

## Introduction

Stroke is the leading cause of disability and death worldwide nowadays [1]. According to the Global Burden of Disease (GBD) Study, the global lifetime risk of stroke from the age of 25 years onward is estimated to have increased from 22.8% in 1990 to 24.9% [2]. Acute cerebral infarction (ACI) is the most common pathological subtype of stroke that accounts for 68%, which caused by blocked or occlusion of a cerebral artery [3]. Patients of ACI, who are typically associated with large blood vessel of the middle cerebral artery (MCA) territory and massive, space-occupying hemispheric infarction, would likely have a devastating prognosis that severe disability or death [4]. Despite intensive care-based treatment, the rate of mortality was up to 80% and most survivors were left with severe neurological disability [5]. Nearly two-thirds of survivors remain severe disability who was completely dependent [modified Rankin scale (mRS) score 4–5] on others with life [6]. The maximal medical treatment, using osmotherapy, diuretic, therapeutic hypothermia, sedative, artificial ventilation and elevated head position for life-threatening, space-occupying brain edema after MMCAI, is still controversial and unsatisfactory [7,8]. Given the limitations of medical therapies, decompressive hemicraniectomy (DHC) has been proposed as a therapeutic alternative that remains a recommendation of the American Heart Association (AHA)/American Stroke Association (ASA) [9]. DHC removes a large part of the skull and opens underlying dura, and normalizes intracranial pressure (ICP) that preserves cerebral blood flow, prevents brain herniation and secondary damage [10]. Most of the classic trials [11–14] shown that DHC could improve survival rate among patients with MMCAI, especially for younger patients (≤60 years) that may be likely to increase the chance of a favorable functional recovery. According to a pooled analysis of three famous and landmark European prospective, randomized controlled trials (RCTs) that conducted in the 2000s (DECIMAL [12], DESTINY [13], HAMLET [14]), current surgical management strategies were based on the results of them [11,14]. Subsequently, the HeADDFIRST [15], Slezins [16], HeMMI [17] RCTs were published that sustained above conclusions. Most of the patients included in these trials are the age of 60 years or younger who can benefit from DHC. As a consequence, it remained unclear whether older patients (>60 years) can also benefit from the DHC. Some results of non-randomized studies indicated that older patientsmay not profit from DHC [18–20]. Therefore, the value of the DHC benefit may be questionable in older patients. Luckily, the cumulative pieces of evidence (ZHAO [21], DESTINY II [22], Li [23]) over the past several years indicated that DHC could substantially reduce death and increase the chance of a favorable functional recovery among older patientsand the upper age to 82 years. Under this background, our systematic review and meta-analysis are to incorporate the results of previous prospective, RCTs and estimates to compare with maximal medical therapy whether DHC among patients with MMCAI improves the survival rate and functional recovery, especially for older patients. Further, it will estimate the optimal time for DHC following the onset of stroke. In brief, the present study aims to provide the best available evidence of clinical practice.

## Materials and methods

### Literature search

Online search from MEDLINE/PubMed, EMBASE, Springer, Cochrane Collaboration database, China National Knowledge Infrastructure (CNKI) database and Wanfang database were performed by two authors (from inception to 1 June 2019). We retrieved the related articles using the following terms: (‘decompressive hemicraniectomy’ OR ‘decompressive craniectomy’ OR ‘decompressive surgery’) AND (‘cerebrovascular disorders’ OR ‘cerebral infarction’ OR ‘ischemic stroke’ OR ‘malignant infarction’ OR ‘middle cerebral artery’) AND (‘randomized controlled trials’ OR ‘randomised controlled trials’) etc. We enlisted the help of a medical librarian to accomplish the search accurately. Details of the search strategy for MEDLINE is shown in Table 1.

**Table 1:** Search strategy used for MEDLINE database

Number	Search terms
#1.	Cerebrovascular disorders/ or brain ischemia/ or carotid artery diseases/ or carotid artery/ or intracranial arterial diseases/ or cerebral arterial diseases/ or infarction, middle cerebral artery/ or exp ‘intracranial embolism and thrombosis’/ or intracranial embolism/ or intracranial thrombosis/ or stroke/ or exp brain infarction/
#2.	((brain or cerebr$ or hemisph$ or intracranial or mca) adj5 (isch?emi$ or infarct$ or emboli$ or thrombo$ or occlus$ or hypoxi$ or apople$)).tw.
#3.	(isch?emi$ adj6 (stroke$ or apoplex$ or cerebral vasc$ or cerebrovasc$ or cva or attack$)).tw.
#4.	1 or 2 or 3
#5.	Decompression/ or decompression surgical/ or neurosurgical procedures/ or hemicraniectomy/or craniotomy/ or trephining/
#6.	(Decompress$ or craniectom$ or craniotom$ or hemi?craniect$ or trepa$ or treph$).tw.
#7.	5 or 6
#8.	4 and 7
#9.	Limit 8 to human
#10.	Randomized controlled trial.pt
#11.	Controlled clinical trial.pt
#12.	Randomized controlled trials.sh.
#13.	Random allocation.sh.
#14.	Double blind method.sh.
#15.	Single blind method.sh.
#16.	10 or 11 or 12 or 13 or 14 or 15
#17.	Limit 16 to human
#18.	9 and 17

This search strategy was modified as required for the other electronic databases.

### Eligible studies

(1) Research design was a prospective, RCTs; (2) Participants:Acute MMCAI patients were confirmed by the WHO diagnostic criteria with threatened cerebral edema or evidence of increased intracranial pressure;(3) Intervention measures: surgery group: DHC. medical treatment group: conservative medical treatment, such as osmotherapy, diuretic, therapeutic hypothermia, sedative, artificial ventilation, and elevated head position etc., and the start time, period, dosage and method of treatment were unlimited; (4) Outcome measures: It reported at least disability or death using the modified Rankin scale (mRS) score (Table 2); (5) Follow-up time: It was at least 6–12 months.

**Table 2:** The modified ranking scale [24]

0. No symptoms
1. No significant disability. Able to carry out all usual activities, despite some symptoms.
2. Slight disability. Able to look after own affairs without assistance,but unable to carry out all previous activities.
3. Moderate disability. Requires some help, but able to walk unassisted.
4. Moderately severe disability. Unable to attend to own bodily needs without assistance, and unable to walk unassisted.
5. Severe disability. Requires constant nursing care and attention, bedridden, incontinent.
6. Dead.

### Literature extraction

**Figure 1:**
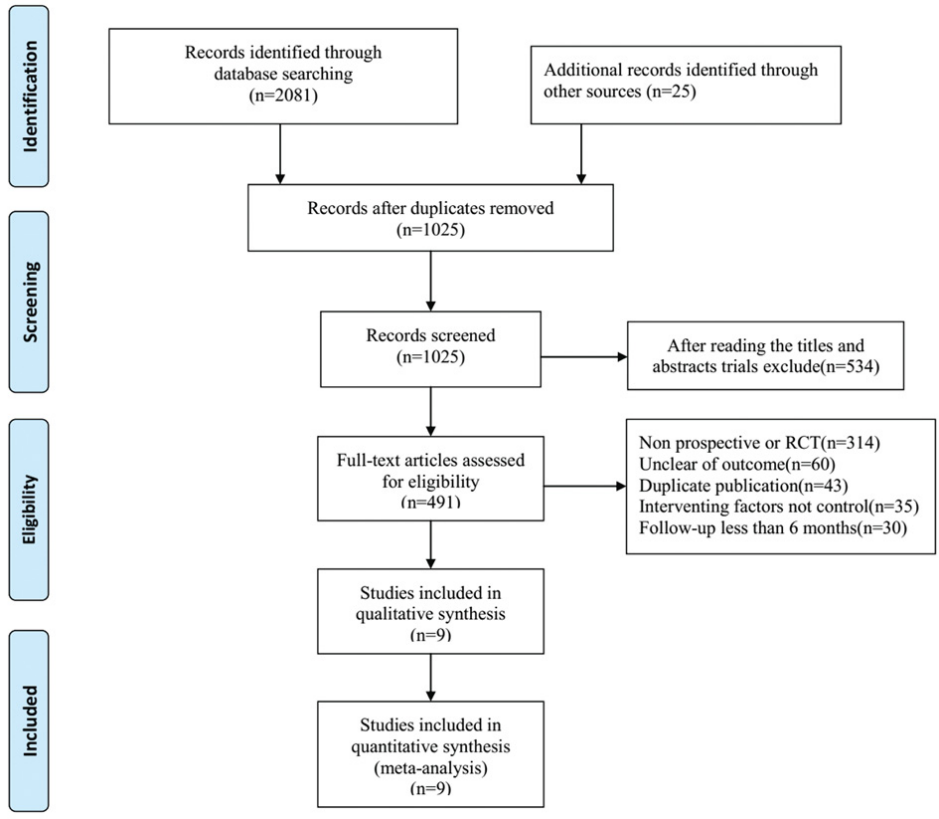
PRISMA flow chart of included studies

All kinds of literature were extracted independently by the first two authors (H. Wei and F.M. Jia) following the Guidelines of the epidemiology of Meta Analysis [25,26]. The third author (X.H. Yin) was able to verify the results if there were differences in the quality of the included studies, as evaluated by the first two authors. Full versions of all relevant kinds of literature were obtained and inspected. Extracted data included the main authors, year of publication, the nation of trial, year of patients, the total number of patients in DHC group and medical treatment group, study type, and outcome indicator etc. The literature selection is presented in the PRISMA flow chart according to the PRISMA guidelines [25,26] (Figure 1).

### Estimation literature of quality and risk of bias assessment

The Jadad scale was used to evaluate the methodological quality of each trial [27–28]. It consists of three parts describing randomization (0–2 points), blinding (0–2 points), and dropouts and withdrawals (0–1 point) in the assessment report of a RCTs. A score of 1 point was given for each condition of the points described. An additional point was given when the study method of randomization and/or blinding was appropriate; however where it was inappropriate, a point was deducted. The Jadad scale ranged from 0 to 5 points. Higher scores (≥3 points) indicated better reporting. The studies were said to be of low quality, if the Jadad score was <2 points, and of high quality, if the score was at least 3. The studies included in our meta-analysis were all Jadad score ≥3 points (Table 3).

**Table 3:** Quality assessment of included studies using the Jadad score

Studies	Randomization	Blinding	Dropouts and withdrawals	Jadad score
DECIMAL [12]	2	2	1	5
DESTINY [13]	2	2	1	5
HAMLET [14]	2	2	1	5
HeADDFIRST [15]	2	2	0	4
Slezins [16]	2	0	1	3
HeMMI [17]	2	2	0	4
Zhao [21]	2	2	1	5
DESTINY II [22]	2	2	1	5
Li [23]	2	0	1	3

Risk-of-bias assessment was performed in accordance with the guidelines outlined in the Cochrane Handbook for Systematic Reviews of Interventions [29]. All studies were independently reviewed by two authors and further assigned a risk of ‘High risk’, ‘Low risk’, or ‘Unclear risk’ to the following: (1) random sequence generation; (2) allocation concealment; (3) blinding of participants and personnel; (4) blinding of outcome assessment; (5) incomplete outcome data; (6) selective reporting.

**Figure 2:**
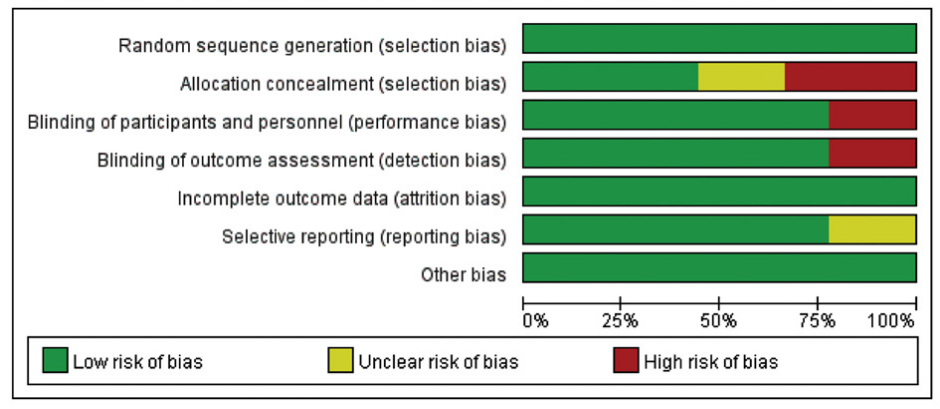
Risk of bias graph

**Figure 3:**
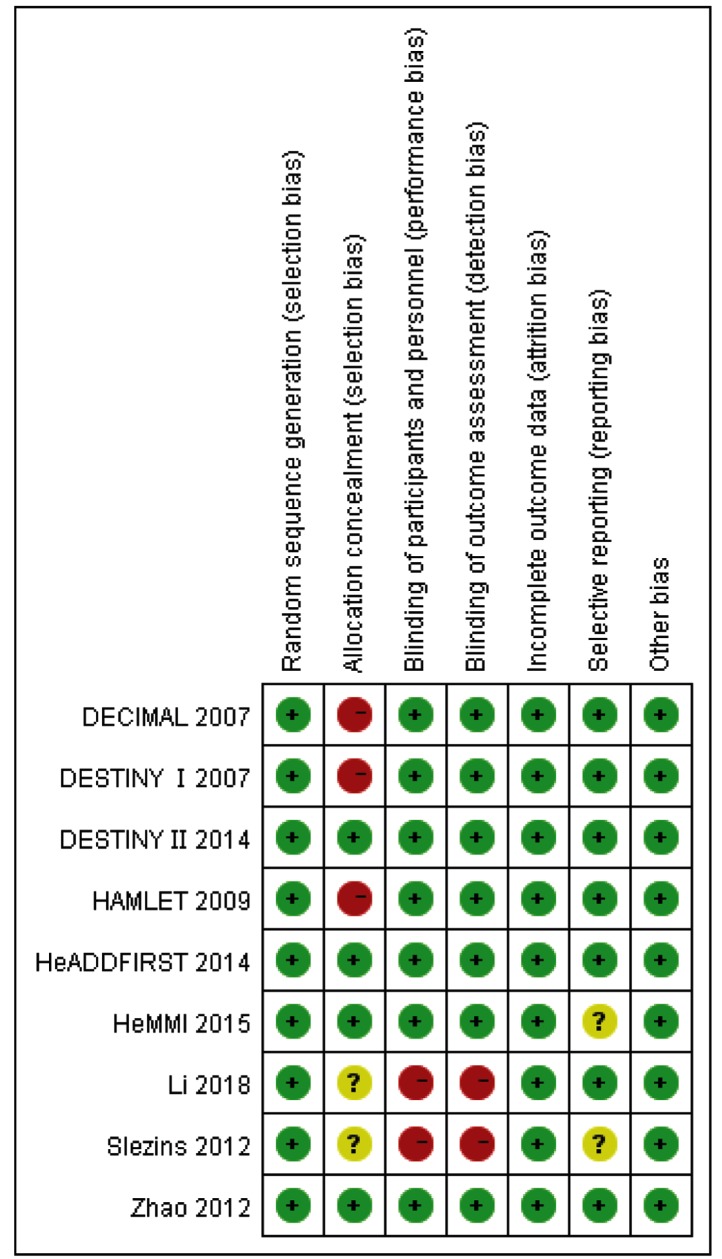
Risk of bias analysis among studies

‘Low risk’ of bias means the description of methods or procedures was adequate, ‘High risk’ of bias means the description of methods or procedures was not adequate or incorrect while ‘Unclear risk’ of bias means there was no description of methods and/or procedures (Figures 2 and 3).

### Statistical analysis

Data meta-analysis was performed using Review Manager (RevMan; Cochrane Collaboration), version 5.3.5 software. The original studies included used RR and 95% CI to assess the association between DHC versus medical treatment among patients for dichotomous outcomes. Statistical heterogeneity across studies was assessed using chi-square test based on *I*^2^ index, which is a quantitative measure of inconsistency across studies. Studies with an *I*^2^ index <25% were considered to have low heterogeneity, if those with an *I*^2^ index of 25–50% were considered to have moderate heterogeneity, and if those with an *I*^2^ index >50% were considered to have high heterogeneity. A random-effects model (REM) was used if there was high heterogeneity (*I*^2^ index>50%) between studies. Otherwise, the fixed-effects model (FEM) was used. The statistical power of meta-analysis was estimated by Power and Precision, version 4 software that we used Alpha = 0.05 and tail = 2 to calculate. A sensitivity analysis was performed to explore the robustness of our analysis. The publication bias was estimated by a funnel plot. All tests were two-sided, with *P* less than 0.05 deemed significant. Since we used only previously published data, we did not need the approval of an ethics committee.

## Results

### Selection and description of studies

Based on search strategies, we identified a total of 2106 published references through electronic searches and hand searches. After reading titles and abstracts, duplications, irrelevant articles, and reviews, we obtained 491 references. After reading the full article, a total of 482 trials were excluded: 314 were not prospective or RCTs, 60 were unclear of outcome, 43 were duplicated publication, in 35 trials the interventing factors were not controlled and in 30 trials follow-up were less than 6 months. Finally, nine prospectives, RCTs were identified in the meta-analysis (Figure 1). It consisted of 210 patients in the DHC group and 215 patients in the medical group. All patients were diagnosed as MMCAI by the diagnostic standard. In the 9 RCTs, the maximum sample size was 109 cases, whereas the minimum sample size was 24 cases. The studies’ and patients’ characteristics are presented in Table 4.

**Table 4:** Study characteristics

Name, publication year and country first author surname	Study design	Duration from symptoms onset to treatment	Age (years) inclusion; median age years (mean)	No. (DHC/Control)	Primary outcome	Jadad
DECIMAL 2007 [12], France, Vahedi	RCTs	Within 30 h	18–55 years 43.5	20/18	mRS as the ordinal outcome at 6 months, 12 months, blinding of outcome assessment	5
DESTINY I 2007 [13], Germany, Jüttler	RCTs	12–36 h	18–60 years; 44.5	17/15	mRS as the ordinal outcome at 6 months, 12 months, blinding of outcome assessment	5
HAMLET 2009 [14], Netherlands, Hofmeijer	RCTs	Within 96 h	18–60 years 50.0	32/32	mRS as the ordinal outcome at 12 months, blinding of outcome assessment	5
HeADDFIRST 2014 [15] U.S.A. and Canada, Frank	RCTs	Within 100 h	18∼75 years 52.3	14/10	Death (mRS = 6) at 21 days, 6 months, blinding of outcome assessment	4
Decompressive Hemicraniectomy 2012 [16], Latvia, Slezins	RCTs	Within 48 h	≥18 years 57.2	11/13	mRS as the ordinal outcome at 12 months, not blinding of outcome assessment	3
HeMMI 2015 [17], Philippina, Chua	RCTs	Within 72 h	18–65 years 50.3	13/11	mRS as the ordinal outcome at 6 months, blinding of outcome assessment	4
Decompressive Hemicraniectomy 2012 [21], China, Zhao	RCTs	Within 48 h	18–82 years 63.5	24/23	mRS as the ordinal outcome at 6 months,12 months, blinding of outcome assessment	5
DESTINY II 2014 [22], Germany, Jüttler	RCTs	Within 48 h	≥60 years; 70	47/62	mRS as the ordinal outcome at 6 months, 12 months, blinding of outcome assessment	5
Decompressive Hemicraniectomy 2018 [23], China, Li	RCTs	Within 48 h	>60 years 65.3	32/31	mRS as the ordinal outcome at 12 months, not blinding of outcome assessment	3

**Figure 4:**
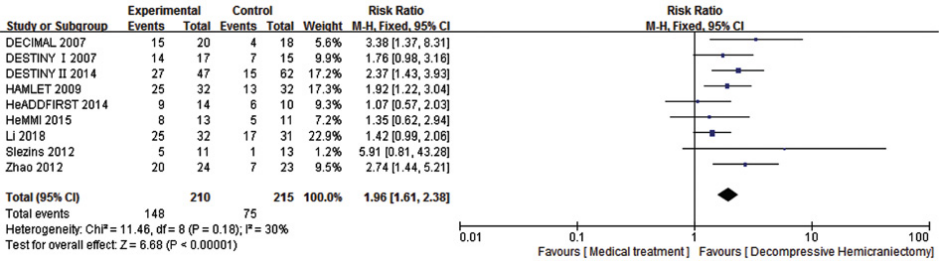
Comparison of DHC versus medical treatment for survival rate (mRS score 0∼5) among patients at 6–12 months

**Figure 5:**
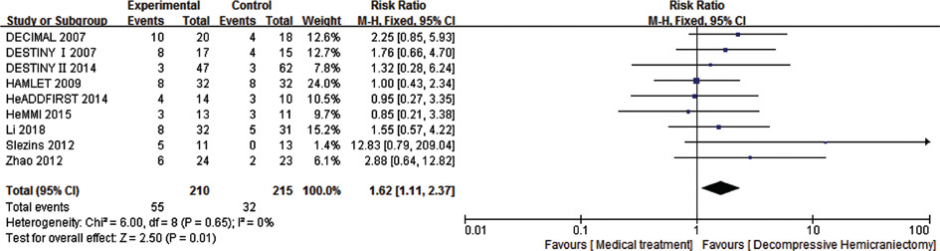
Comparison of DHC versus medical treatment for the favorable functional recovery (mRS score 0–3) among patients at 6–12 months

For the analysis of survival rate (mRS score 0–5) at 6–12 months, 9 studies and 425 patients were included. After the treatment at follow-up (6–12 months), compared with the medical treatment group, DHC was associated with statistically significant increase survival rate (RR: 1.96, 95%CI 1.61–2.38, *P* < 0.00001) with the statistical power of 100% and favorable functional recovery (RR: 1.62, 95%CI 1.11–2.37, *P* = 0.01) with the statistical power of 80% (Figures 4 and 5).

### Subgroup analysis

A subgroup analysis was performed based on age. Separately 4 studies with 152 patients age ≤60 years and 3 studies with 201 patients aged >60 years showed that survival rate (mRS score 0–5).

**Figure 6:**
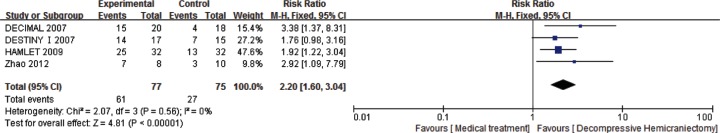
Comparison of DHC versus medical treatment for survival rate(mRS score 0∼5) among patients of age ≤60 years at 6–12 months

First, among patients of age ≤60years, compared with the medical treatment group, DHC was associated with a statistically significant increase survival rate (RR: 2.20, 95%CI 1.60–3.04, *P* < 0.00001) with the statistical power of 100% (Figure 6).

**Figure 7:**
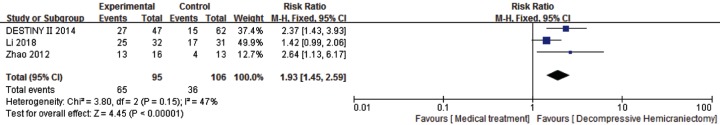
Comparison of DHC versus medical treatment for survival rate (mRS score: 0–5) among patients of age >60 years at 6–12 months

Second, among patients of age >60 years, compared with the medical treatment group, DHC was associated with a statistically significant increase survival rate (RR: 1.93, 95%CI 1.45–2.59, *P* < 0.00001) with the statistical power of 100% (Figure 7).

**Figure 8:**
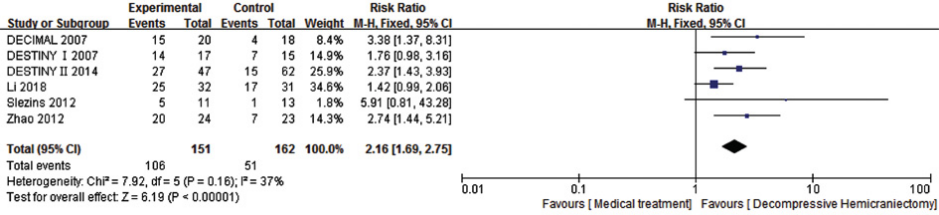
Comparison of DHC versus medical treatment for survival rate (mRS score: 0–5) among patients who were performed DHC within 48 h of onset stroke at 6–12 months

Another subgroup analysis was performed based on DHC time within 48 h from the onset of stroke. Six studies with 313 patients were included. It showed that compared with the medical treatment group, DHC within 48 h from the onset of stroke was associated with a statistically significant increase survival rate (RR: 2.16, 95% CI 1.69–2.75, *P* < 0.00001) with the statistical power of 100% (Figure 8).

### Sensitivity analysis

We used the method of removing item by item to test the stability of meta-analysis, and the results showed that there had been no noticeable change on any of the outcomes. The difference between the REM and FEM may have influenced the outcomes. Therefore, we used different statistical models to pool the data for the mRS score 0–5, mRS score 0–3, age ≤60 years, age >60 years and mRS score 0–5 within 48 h from the onset of stroke. No observable change in any of the outcomes was found (Table 5).

**Table 5:** Results of sensitivity analysis

Study type	Studies (*n*)	DHC group (*n*)	Control group (*n*)	χ2	d*f*	*I*^2^, %	*P* value	Analysis model	RR (95%CI)	*P* value
mRS 0–5	9	210	215	11.46	8	30	0.18	Fixed	1.96 (1.61–2.38)	<0.00001
								Random	1.84 (1.44–2.35)	<0.00001
mRS 0–3	9	210	215	6.00	8	0	0.65	Fixed	1.62 (1.11–2.37)	0.01
								Random	1.50 (1.02–2.21)	0.04
Age ≤60	4	77	75	2.07	3	0	0.56	Fixed	2.20 (1.60–3.04)	<0.00001
								Random	2.10 (1.53–2.88)	0.003
Age >60	3	95	106	3.80	2	47	0.15	Fixed	1.93 (1.45–2.59)	<0.00001
								Random	1.91 (1.25–2.93)	<0.00001
mRS 0–5	6	151	162	7.92	5	37	0.16	Fixed	2.16 (1.69–2.75)	<0.00001
≤48 h								Random	2.10 (1.52–2.90)	<0.00001

### Publication bias

**Figure 9:**
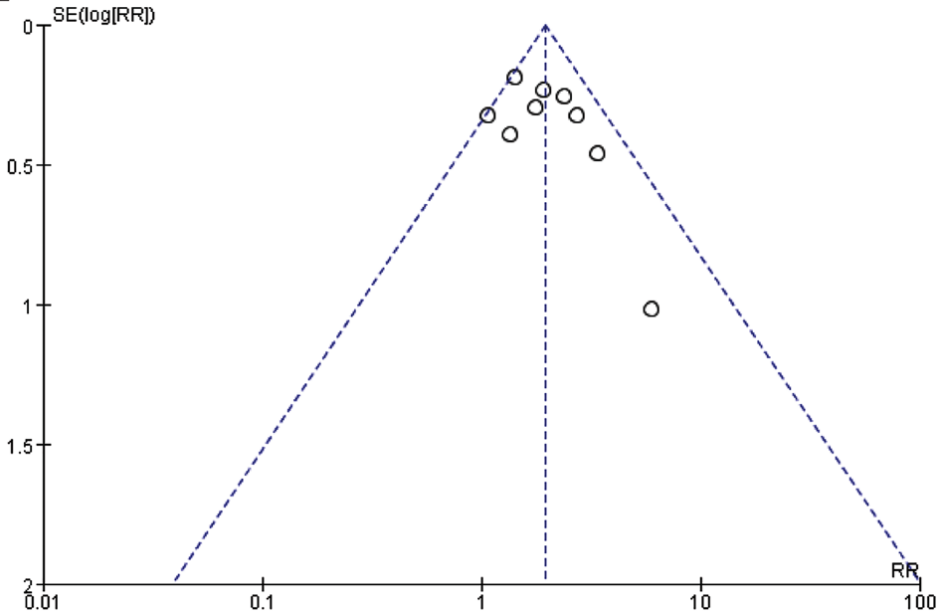
The funnel plots of publication bias

A funnel plot was used to evaluate the publication bias. A total of nine RCTs included in the funnel plot of the survival rate. As shown in Figure 9, the funnel plots did not demonstrate any obvious asymmetry.

## Discussion

### Main findings

Cerebral edema can generate and develop after ACI, especially in MMCAI. Owing to the rigid nature of the skull, escalating cerebral edema leads to increase ICP that causes a reduction in cerebral perfusion pressure, cerebral blood flow, and oxygenation. These effects, if not interrupted, can lead to brain herniation and death. Because of the current limitations of medical therapy in controlling cerebral edema, DHC, a procedure whereby part of the skull is removed and the underlying dura is opened, is attractive for the management of deteriorative cerebral edema. DHC can provide additional space for the swollen brain, which can decrease the risk of ICP elevation and herniation. Recommendations of current guidelines remove a bone flap of at least 12 cm, and possibly up to 13–14 cm in some patients that can sufficiently expose the frontal, temporal, and parietal lobe. Although the passing of 100 years since the first description of DHC in 1901, the role of this surgery inpatient management continues to be debated. The exact indications for DHC, effects of DHC on long-term functional outcome, especially in patients >60 years and optimal time of DHC treatment remain unclear. Our study is aimed to enroll the high-quality RCTs using for illuminating the controversies.

After strictly systematic review and meta-analysis, the results of our study, which included 9 RCTs comprising of 425 patients, indicated that DHC appeared to be helpful and effective for MMCAI in survival rate and favorable functional recovery. The included studies are similar to other recent systematic reviews of this question (DHC vs medical treatment) [6,30–31]. First, it demonstrated that DHC for MMCAI with threatened edema results in large increase survival rate (mRS score 0–5). Second, it showed that DHC increased the likelihood of being a favorable functional outcome survivor (mRS score 0–3) when compared with the maximal medical treatment in patients of age ≤60 years or >60 years. Third, subgroup analysis included 6 studies that DHC increased the likelihood of being a survivor (mRS score 0–5) when compared with the maximal medical treatment at the time of surgery within 48 h from the symptom onset. Our results are consistent with those of other previous reviews [6,30–31] of RCTs involved in DHC after MMICA stroke. None of the previous reviews, however, have included all 9 prospectives, RCTs to respectively analyze of age ≤60 or >60 years.

### Strengths and limitations

Owing to age is an important predicting factor for prognosis of stroke, previous studies (DECIMAL, DESTINY, and HAMLET) included patients only age ≤60 years and numerous non-randomized reports suggesting that elderly patients may not profit from DHC. With the ZHAO, DESTINY II and Li studies were published that included patients age >60 years and the upper age to 82 years, these indicated that patients of >60 years can also profit from DHC. So our subgroup meta-analysis respectively patients of age ≤60 or >60 years and results of analysis that both age ≤60 or >60 years all can have a good prognosis with DHC that compared with medical treatment. Previous studies [32] have found that the progression of cerebral edema after ACI ranges between 2–5 days: while 68% of patients early exhibit clinical deterioration within 48 hours from the symptom onset, especially in MMCAI.

DHC surgery was performed before signifcant neurological deterioration was very important. Therefore, subsequent guidelines recommended DHC surgery be pursued within 48 h from the onset of stroke [33].

So, our subgroup meta-analysis also verified that the time of surgery within 48 h from the onset of stroke could improve survival rate. Dasenbrock [34] study enrolled 1301 of ACI patients and showed that when surgery was pursued after 48 h from the onset of stroke, it increases poor outcome (OR = 1.12, 95%CI 1.02–1.23) than surgery within 48 h. Hence, more early surgery preformed, more favorable recovery gained. However, performing DHC before herniation may be the most important present consideration.

Our meta-analysis had several limitations. First, the RCTs included in the present study were of limited sample size and studies number, 6 of 9 studies included less than 50 patients. Second, the follow-up duration in 2 studies was less than 12 months. Third, the time of DHC surgery in 3 studies was performed more than 48 h from the onset of stroke, which may affect the effectiveness of DHC. And owing to these 3 studies had a small sample size, we did not meta-analysis the prognosis of DHC that was performed more than 48 h of onset symptoms. Finally, several primary studies, for example, high risks of bias problems included lack of allocation concealment, lack of blinding of participants and personnel, and lack of blinding of outcome assessment.

## Conclusion

The results of our study indicated that DHC could increase survival rate and favorable functional recovery among patients age ≤60 or >60 years. The optimal time for surgery may be within 48 h from the onset of stroke. More RCTs are necessary to further prove the effects of DHC, especially in those with age >60 years or severe disability.

## Abbreviations

ACI: acute cerebral infarction
CNKI: China National Knowledge Infrastructure
DHC: decompressive hemicraniectomy
FEM: fixed-effects model
GBD: Global Burden of Disease
ICP: intracranial pressure
MCA: middle cerebral artery
MMCAI: malignant middle cerebral artery infarction
mRS: modified Rankin scale
RCT: randomized controlled trial
REM: random-effects model
